# Using an individual-based model to assess common biases in lek-based count data to estimate population trajectories of lesser prairie-chickens

**DOI:** 10.1371/journal.pone.0217172

**Published:** 2019-05-17

**Authors:** Beth E. Ross, Daniel S. Sullins, David A. Haukos

**Affiliations:** 1 U.S. Geological Survey, South Carolina Cooperative Fish and Wildlife Research Unit, Department of Forestry and Environmental Conservation, Clemson University, Clemson, South Carolina, United States of America; 2 Kansas State University, Division of Biology, Manhattan, Kansas, United States of America; 3 U.S. Geological Survey, Kansas Cooperative Fish and Wildlife Research Unit, Manhattan, Kansas, United States of America; Bowling Green State University, UNITED STATES

## Abstract

Researchers and managers are often interested in monitoring the underlying state of a population (e.g., abundance), yet error in the observation process might mask underlying changes due to imperfect detection and availability for sampling. Additional heterogeneity can be introduced into a monitoring program when male-based surveys are used as an index for the total population. Often, male-based surveys are used for avian species, as males are conspicuous and more easily monitored than females. To determine if male-based lek surveys capture changes or trends in population abundance based on female survival and reproduction, we developed a virtual ecologist approach using the lesser prairie-chicken (*Tympanuchus pallidicinctus*) as an example. Our approach used an individual-based model to simulate lek counts based on female vital rate data, included models where detection and lek attendance probabilities were <1, and was analyzed using both unadjusted counts and an N-mixture model to compare estimates of population abundance and growth rates. Using lek counts to estimate population growth rates without accounting for detection probability or density-based lek attendance consistently biased population growth rates and abundance estimates. Our results therefore suggest that lek-based surveys used without accounting for lek attendance and detection probability may miss important trends in population changes. Rather than population-level inference, lek-based surveys not accounting for lek attendance and detection probability may instead be better for inferring broad-scale range shifts of lesser prairie-chicken populations in a presence/absence framework.

## Introduction

Monitoring fish and wildlife populations can be challenging. While managers are often interested in monitoring the underlying state of the system (e.g., abundance), error in the observation process might mask underlying population changes. Imperfect detection, availability for sampling, and heterogeneity in abundance can all mask underlying changes of abundance [1,2]. When using counts of abundance to estimate changes in population growth rate over time, sampling issues can cause biased inference. For example, if detection probability is not properly incorporated into estimates of population growth rate, even a small change (4–8%) in detectability between two treatments can lead to a 50–90% increase in committing a Type 1 error (detecting a difference when none exists; [3]).

To determine the reliability of a monitoring program or survey to detect changes in the underlying state process of a species, we conducted a simulation study (also referred to as a “virtual ecologist” approach [4,5]). The virtual ecologist approach simulates an underlying population based on known, or at least empirically estimated, variables and an observation process. For example, the virtual ecologist approach could simulate how varying detection ultimately affects population growth rate estimates. The resulting data are then analyzed in the same manner that field data would be analyzed. This approach allows researchers to evaluate their assumptions about the monitoring program at multiple steps (e.g., process changes versus changes in observation error), and assess where assumptions may differ from reality.

Lekking avian species present a unique population monitoring problem. While female survival and reproduction rates are typically used to model population demography [6], male-based lek surveys are commonly used to monitor population change of lekking species [7]. Monitoring only the male portion of the population assumes changes in abundance of males attending leks represents changes in overall population abundance, which may not always hold true [8]. While it is easiest to monitor male abundance on leks to track population abundance, annual population growth rates calculated from lek counts may be biased high relative to annual population growth rates calculated from demographic rates [8,9]. Differences between population growth rates calculated with demographic rates or lek counts are likely due to variation in lek attendance within and among seasons, including relatively long-term lag effects in male attendance on leks. Some males may not attend leks in a given year, or form unobserved satellite leks away from more established leks [8,10]. Furthermore, due to lek fidelity by males, local extinctions of females may not be detected for several years. Although males attending larger leks may be more conspicuous, males that do not attend historically occupied leks may go undetected during traditional roadside surveys [11,12]. Observing these transient individuals through traditional survey methods is difficult and can lead to bias in population estimates as temporary emigration and detection probability are confounded [13]. Furthermore, annual variation in abundance is not independent of lek attendance, temporary emigration, and detection probability [8]. For example, the rate of lek attendance is likely affected by abundance of males due to increased competition on leks among first-year or less successful males in years with greater abundance. Decreased attendance of leks during years of high population abundance may bias estimates of population change as mortality of males attending leks is undetected if replacement males are available in the population. Further, small satellite leks are more likely to form when population abundance is high and less likely when it is low [10,14]. Thus, if a survey misses satellite leks, a population might appear to be stable when it is in fact fluctuating.

We developed a simulation approach to better understand how well lek count surveys estimate changes in population growth rate through time. Our work was motivated by the apparently contradictory findings of recent studies on lesser prairie-chickens (*Tympacnuchus pallidicinctus*; [15,16]). While one study found a stable population over the last several years based on male lek counts (2012–2016; [15]), the other predicted a high likelihood of extirpation of the lesser prairie-chicken from large portions of its range based on female vital rates [16]. Ultimately, we were interested in determining if, due to observation error and variation in lek attendance, lesser prairie-chicken populations could exhibit a stable population growth rate based on lek-count data but have decreasing population growth rates when computed from vital-rate data (e.g., adult survival and fecundity). Our simulation approach used an individual-based model to simulate lek counts based on vital rate data, including models where detection probability and lek attendance were less than 1, and was analyzed using an N-mixture model to estimate population abundance and growth rates [17]. In particular, we were interested in better understanding how lek attendance rates and detection probability affect estimates of population growth rates.

## Materials and methods

### Study system: The lesser prairie-chicken

For our study, we focused on a lek-mating grouse species, the lesser prairie-chicken, which persists in the southwestern Great Plains of Kansas, Colorado, New Mexico, Texas, and Oklahoma. The lesser prairie-chicken is of conservation concern because of habitat loss and population declines throughout its range [18,19]. Recent population-level studies based on lek-count survey data estimated a relatively stable population over the last 5 years [15,20] or stable with decreases through a 2012–2013 drought event [21]. Alternatively, studies using vital rate data from the same population (i.e., adult survival and fecundity) incorporated into a population viability analysis estimated declines in the population over the last decade with finite population growth rates <1 [16, 22]. Given such discrepancies in inference between approaches, better understanding of the limitations and strengths of current monitoring protocols is needed.

To address potential discrepancies between population growth rates based on lek counts and vital rate data, we developed a virtual ecologist approach to simulate monitoring lesser prairie-chicken populations. We used three steps in our framework: 1) simulate the underlying abundance process using an individual-based model (“Process Model,” below), 2) simulate the observation process on leks (“Observation Model”), and 3) analyze our resulting abundance estimates for comparison to our known simulated values (“Analysis”).

### Process model: Description of individual-based model

The individual-based model (IBM) was based on demographic estimates from literature on lesser prairie-chickens in Kansas ([22–25]; Table 1). Currently, >90% of the extant estimated abundance of lesser prairie-chickens occurs in ecoregions represented in Kansas [20]. We initialized our simulation with 100 individuals, 50 males and 50 females, at 300 different sites (30,000 individuals total, approximately the estimated range-wide abundance of lesser prairie-chickens during 2017; [20]). The age distribution was 60:40 after-second year (ASY): second year (SY; [24]) for both sexes. We ran the IBM for 25 years. We ran a “base” model of the IBM with the assumption of perfect detection probability (i.e., no simulated observation process) and lek attendance by all males. We expand on these two assumptions with different scenarios below (“Model Scenarios”). Within the IBM, we simulated four stages of the life cycle: 1) reproduction, 2) annual mortality, 3) aging, and 4) lek attendance. Code to implement the IBM is available in S1 File.

**Table 1:** Values of demographic parameters used to simulate individual-based models for lesser prairie-chickens in Kansas.

Parameter	Mean	SD	Data Source
Male adult survival	0.45	0.06	[11]
Female adult survival: breeding	0.49	0.05	[22]
Female adult survival: non-breeding	0.73	0.04	[25]
Nesting propensity	0.95	0.04	[22]
Clutch size_1_	10.80	2.17	[22]
Nest success_1,SY_	0.47	0.04	[22]
Nest success_1,ASY_	0.44	0.05	[22]
Renest	0.33	0.15	[22]
Clutch size_2_	8.17	2.02	[22]
Nest success_2,SY_	0.50	0.01	[22]
Nest success_2, ASY_	0.33	0.08	[22]
Hatch	0.93	-	[24]
Chick survival	0.26	0.01	[22]
Juvenile survival	0.54	0.09	[24]
SY lek attendance	0.60	0.10	[10]
ASY lek attendance	0.80	0.10	[10]
Female lek attendance	0.30	0.10	-

*Reproduction*–We simulated fecundity for ASY and SY individuals, or the number of offspring per female, *F*_*j*_ for *j* = ASY and SY, as

$$F_{j}=[Nest\;Propensity\times Clutch_{1}\times Nest_{1,j}+(1‐Nest_{1,j})\times Renest\times Clutch_{2}\times Nest_{2,j}]\times Chick\;Surv\times Juvenile\;Surv\times 0.5\times Hatch$$

where *Nest Propensity* was the probability of a female initiating a nest, *Clutch*_1_ was the number of chicks fledged from the first nest, *Nest*_*1*,*j*_ is the probability of nest success, *Renest* is the probability of renesting, *Clutch*_*2*_ is the number of chicks fledged from a second nest, *Nest*_*2*,*j*_ is the probability of nest success for a second nest, *Chick Surv* is the probability of chick survival from hatch until 60 days after hatch, *Juvenile Surv* is the probability of a juvenile surviving from 60 days after hatch until the following breeding season, and *Hatch* is the probability of hatch rate for an individual egg (Table 1). We assumed a 50/50 female/male ratio for offspring. Our model for reproduction assumes no sex-specific differences in chick and juvenile survival.

*Annual Mortality*–Mortality differed by sex, and female survival differed between breeding and non-breeding season (Table 1).

*Aging*–We allowed individuals to age by one year.

*Lek attendance*–We selected a proportion of the total individuals at a site to attend leks during each year. Our definition of lek attendance was based on annual attendance rather than temporal emigration onto or off a lek between survey periods. We simulated different proportions of individuals attending leks by age and sex on an annual basis. SY males attended leks less than ASY males (60% vs 80% respectively; Table 1; [11]). Females are occasionally included in lek counts, although we are aware of no previous studies quantifying the proportion of counts based on females, so we used a probability of annual lek attendance of 30%. Variation in lek attendance for all individuals was set at SD = 0.1 to account for uncertainty in estimates of lek attendance rates from the literature.

To assess how lek attendance within a lek complex affects population growth rate estimates, we also simulated a rate of lek attendance for males dependent on total population abundance. The mean density-based lek attendance, γ, was defined as

$$\begin{array}{ccc} \gamma =1 & \mathrm{if} & K>N_{tot}\\ \gamma =\frac{K}{N_{tot}} & \mathrm{if} & K<N_{tot} \end{array}$$

where *K* is the carrying capacity of a lek and *N*_*tot*_ is the total population abundance. Variation in lek attendance rate was set at SD = 0.1, as in the base model. We set the carrying capacity of a lek at 25 male individuals but did not include the number of females in *N*_*tot*_. We contrasted the effects of density-varying lek attendance with the base model assuming no density variation to estimate the effect on the estimate and variance of λ. We also estimated the absolute change of λ to change in carrying capacity.

### Observation model: Monitoring of leks

Two repeated counts of leks were simulated within the individual-based model and count data were generated from these repeated counts by using a binomial distribution where *N*_*OBS*,*i*,*t*_ ~ Binomial(*N*_*LEK*,*i*,*t*_, *p*). We then used different methods of analyzing the repeated count data (below in “Analysis”) and scenarios with different forms of detection error (Table 2). Previous work estimated the detection probability of lesser prairie-chickens from road-based transect surveys, but did not quantify the effect of observer, time of day, or other environmental factors that may affect detection probability across the range of the lesser prairie-chicken ([19,26]). Additionally, given the nature of surveys for leks, there are two components to the detection process: detecting the lek and correctly identifying individuals on the lek. In our simulations, we assumed perfect detection of each lek, and defined the probability of detection as the product of the probability of detecting individuals on the lek and the probability of temporary absence (birds leaving leks between surveys). To quantify the effect of detection probability <1 on estimates of λ, we used three levels of constant detection probability: low (*p* = 0.25), intermediate (*p* = 0.375), and high (*p* = 0.50). The intermediate level of detection probability was based on the average detection probability from previous work [19,26]. We also created three scenarios where detection probability randomly varied between 0.25 and 0.75 for each site, year, or year and site. Additionally, as there are several reasons for decreasing trends in detection probability through time (e.g., increasing noise pollution or observer variation; [27]), we developed a simulation that began with high detection (0.75) and decreased each year by 0.03 to 0.25. We estimated the sensitivity of our estimate of λ to changes in detection probability.

**Table 2:** Scenarios run in individual-based models for lesser prairie-chickens.

Scenario #	Scenario	*p*	Lek attendance
1	Perfect lek attendance and perfect detection	1.0	1.0
2	Proportion of males attend, but not density-based lek attendance	1.0	0.6/0.8
3, 3N	Perfect lek attendance, p<1	0.5	1.0
4, 4N	Density-based lek attendance	0.5	K/N
5, 5N	Detection probability <1, fixed	0.25	0.6/0.8
6, 6N	Detection probability <1, fixed	0.375	0.6/0.8
7, 7N	Detection probability <1, fixed	0.5	0.6/0.8
8, 8N	Randomly varied detection (site only)	0.25–0.75	0.6/0.8
9, 9N	Randomly varied detection (year only)	0.25–0.75	0.6/0.8
10, 10N	Randomly varied detection (site + year)	0.25–0.75	0.6/0.8
11, 11N	Trend in detection	0.75 decreasing to 0.25	0.6/0.8
12, 12N	Density-based lek attendance and p <1	0.5	K/N
13, 13N	Density-based lek attendance and p randomly drawn (year only)	0.25–0.75	K/N

### Analysis: State-space and N-mixture models

We evaluated the bias of our simulations using two methods, one that accounted for imperfect detection and one that did not. In both cases, we used a latent model for abundance

$$N_{i,t}=N_{i,t-1}\lambda_{i,t}$$

$$\lambda_{i,t}∼\mathrm{Gamma}(r,\beta )$$

where the annual population growth rates (*λ*_*i*,*t*_) vary by site and time but are randomly drawn from a Gamma distribution with $r={\bar{\lambda }}^{2}/\sigma_{\lambda }^{2}$ where $\bar{\lambda }$ is the long-term average population growth rate with variance $\sigma_{\lambda }^{2}$, and $\beta =\bar{\lambda }/\sigma_{\lambda }^{2}$. We used the priors $\bar{\lambda }$ ~ Gamma(0.10,0.10) and *σ_λ_* ~ Gamma(3,1).

### State-space model

The observation model for the state-space model was specified as

$$y_{i,t}∼\mathrm{Poisson}(N_{i,t})$$

where *y*_*i*,*t*_ were count data with the maximum count recorded at each site and year. We simulated the maximum count by taking the greater count from two detection occasions, each with detection probability *p*. The ***N*** were estimated from Eq (1).

### N-mixture model

In the second approach to our analysis, we used a Bayesian N-mixture model [17,26,28], which allowed us to quantify the ability to detect long-term trends in abundance while also accounting for detection probability. As current monitoring for lesser prairie-chickens does not account for lek attendance rates <1, we did not include this in our N-mixture model, although this violates the closure assumption of the model. We therefore assumed population closure for N-mixture model results. We specified the count data, y_*i*,*j*,*t*_ for sites *i*, occasion *j*, and time *t* for the N-mixture model as

$$y_{i,j,t}∼\mathrm{Binomial}(X_{i,t},p_{i,t})$$

We then modeled the underlying mean abundance as *X*_*i*,*t*_ ~ Poisson(*N*_*i*,*t*_) where *N*_*i*,*t*_ was estimated from Eq (1). We assigned vague priors of *p*_*i*,*t*_ ~ Beta(1,1).

### Evaluation

We calculated the bias in estimated $\bar{\lambda }$ relative to the true value of $\bar{\lambda }$ to evaluate the ability of the state-space model and N-mixture model to capture long-term trends in population abundance. For each of 100 simulations, *s*, we calculated bias as $\theta_{s}={\bar{\lambda }}_{s}^{sim}-{\bar{\lambda }}_{s}^{true}$. We calculated ${\bar{\lambda }}_{s}^{true}$ as

$${\bar{\lambda }}_{s}^{true}=\sum_{i=1}^{I}\frac{\left(\prod_{t=1}^{T}N_{s,i,t}/N_{s,i,t-1}\right)}{I}$$

the averaged geometric mean of the annual population growth rate (*λ*_*s*,*i*,*t*_ = *N*_*s*,*i*,*t*_/*N*_*s*,*i*,*t-1*_). We calculated the value for ${\bar{\lambda }}_{s}^{sim}$ by taking the mean over all Markov Chain Monte Carlo (MCMC) iterations. The mean and 95% quantiles for *θ*_s_ were then calculated over 100 simulations for evaluation.

## Results

**Fig 1:**
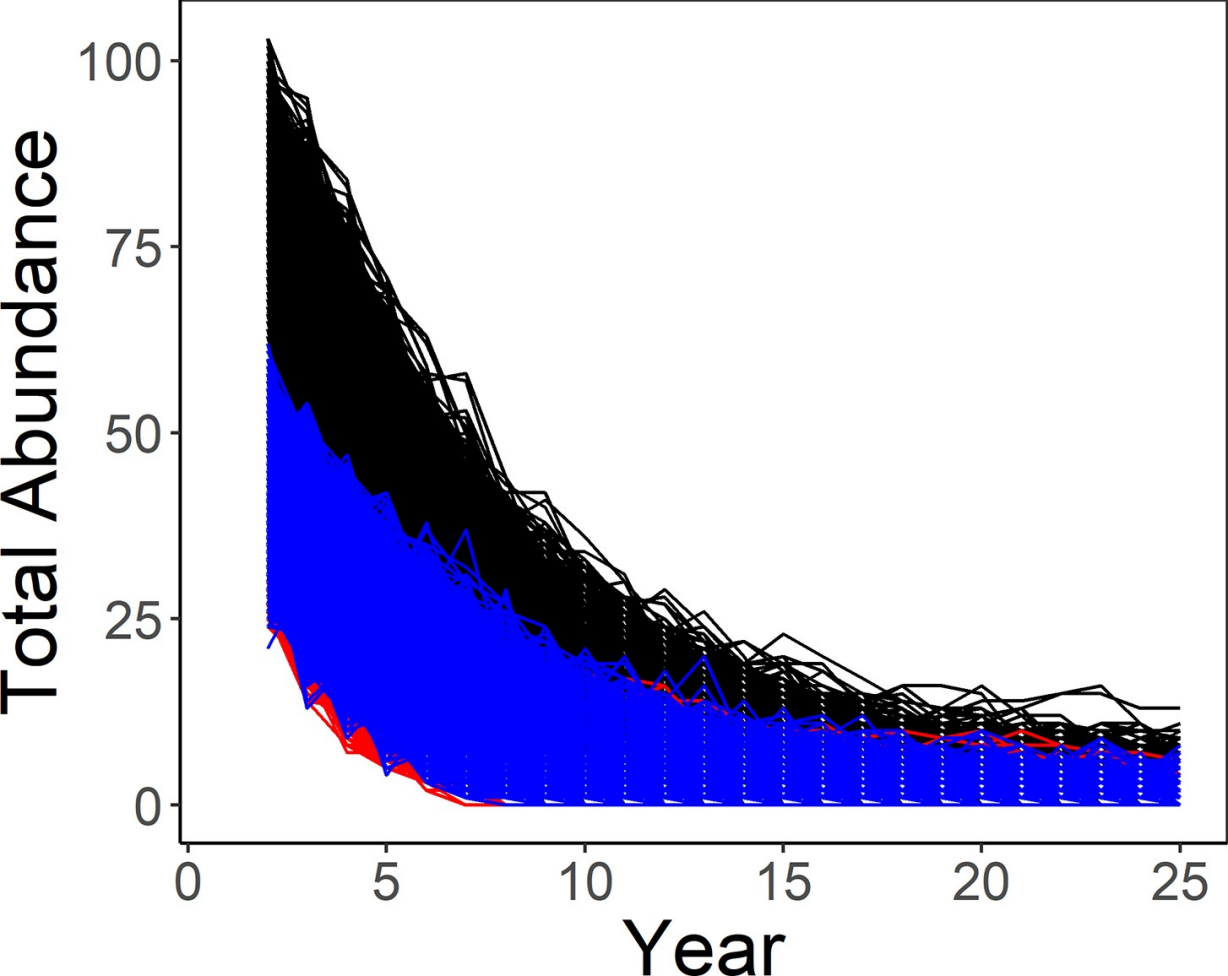
Simulated total lesser prairie-chicken abundance. Simulated (Scenario 2) total lesser prairie-chicken population abundance (black) from 100 individual-based model simulations of 300 sites with male abundance (blue) and female abundance (red).

In 29,557 of 30,000 simulations (100 simulations at 300 sites) of our base model (lek attendance probability and detection probability = 1; Scenario 1), we reached population extinction over a 25-year period with an initial population of 100 total birds (50 females, 50 males). All simulations ended with fewer than 10 birds, likely a pseudo-extinct population (Scenario 2; Fig 1).

### Estimates not adjusted for detection probability with N-mixture model

**Fig 2:**
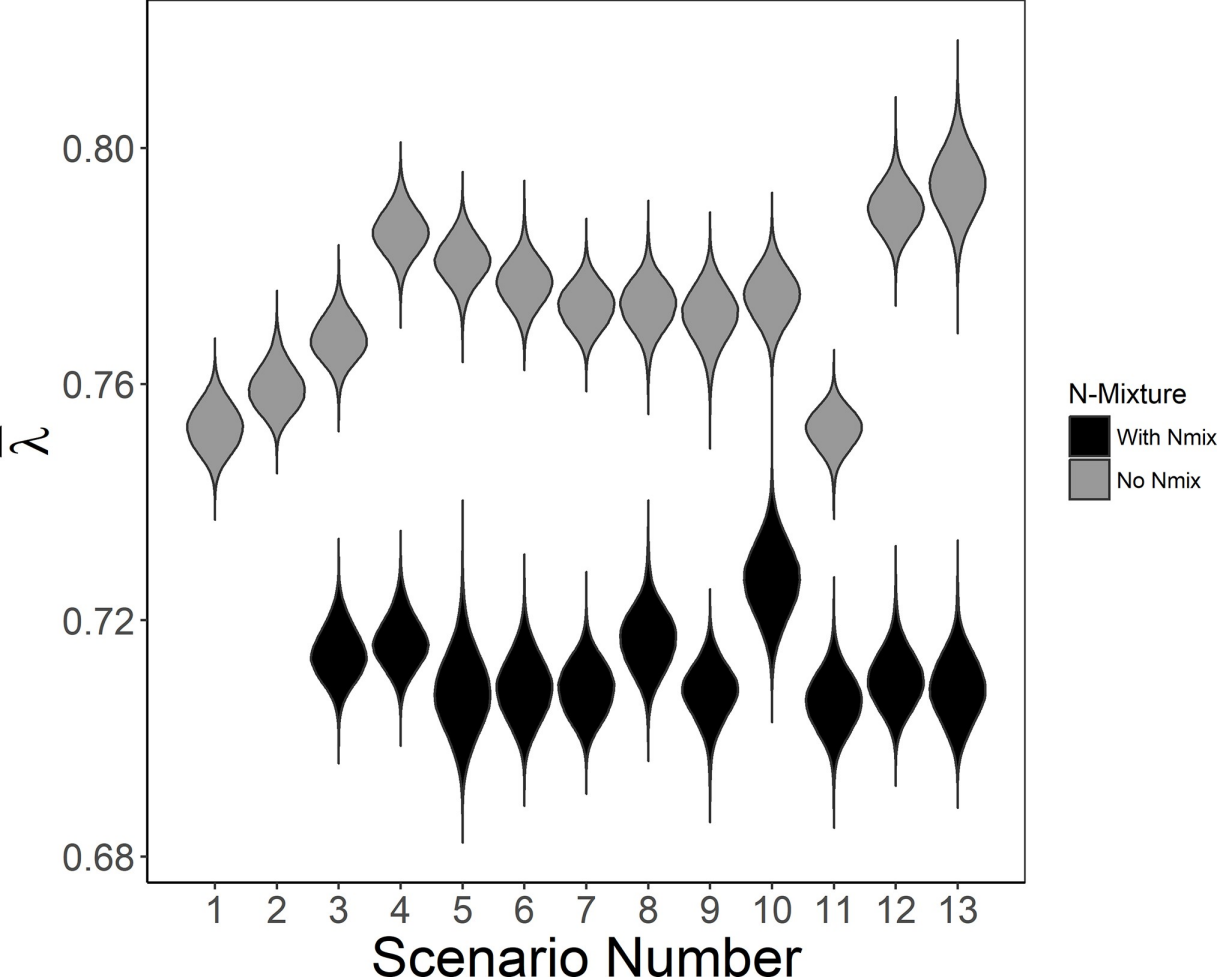
Mean long-term population growth rates ($\bar{\lambda }$) from simulated lek counts of lesser prairie-chickens based on individual-based models with different scenarios (see Table 2). Each violin plot represents all Markov Chain Monte Carlo (MCMC) samples from the 100 simulations combined for the models without the N-mixture model (“No N-mix”, Scenarios 1–13) and with the N-mixture model (“With N-mix”, Scenarios 3N-13N).

**Fig 3:**
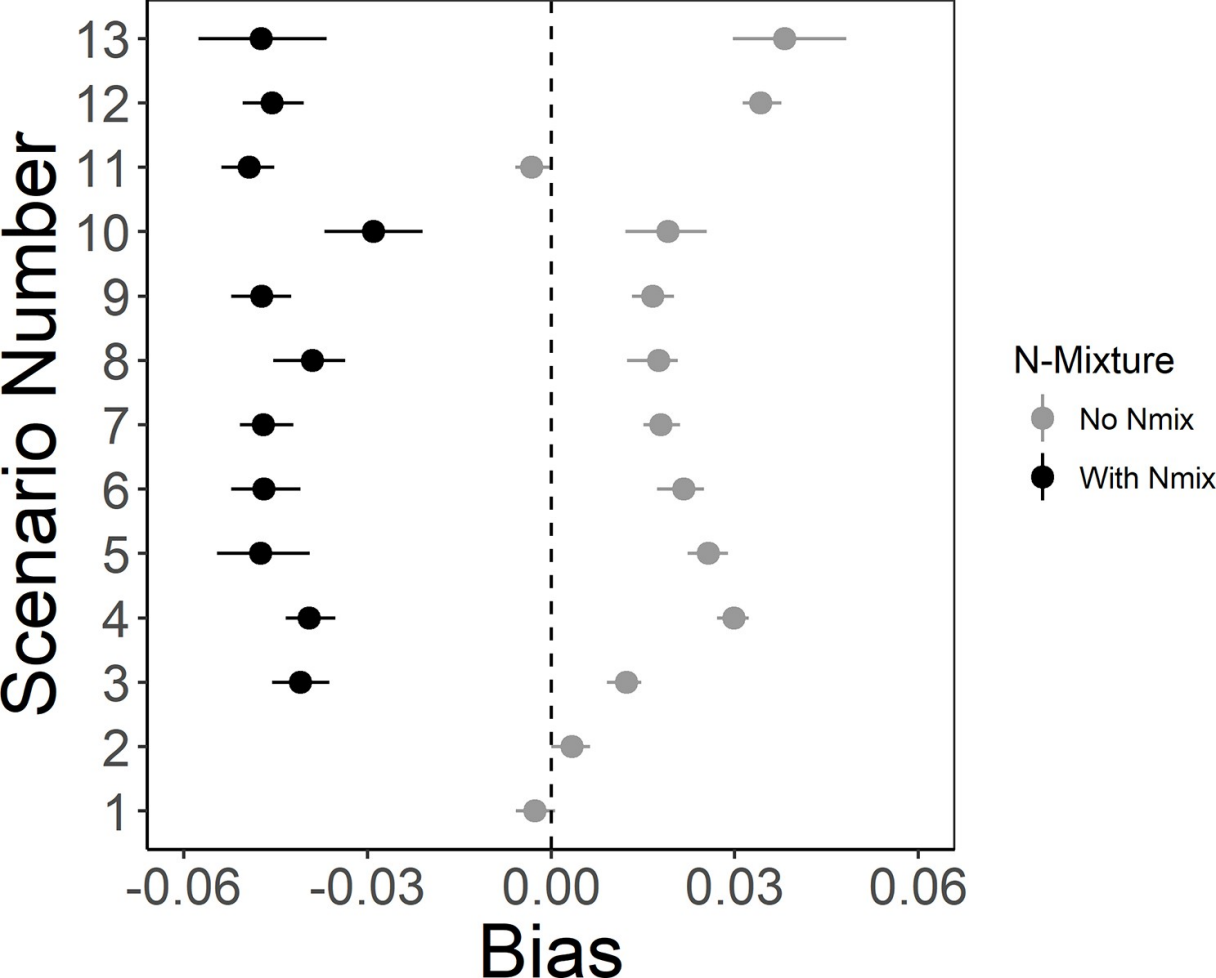
Bias of mean long-term population growth rates ($\bar{\lambda }$) from simulated lek counts of lesser prairie-chickens based on individual-based models (see Table 2). The bias between the mean estimated $\bar{\lambda }$ for each simulation and the true value for that simulation. Points represent the mean bias over all 100 simulations and whiskers represent the 95% quantiles.

**Fig 4:**
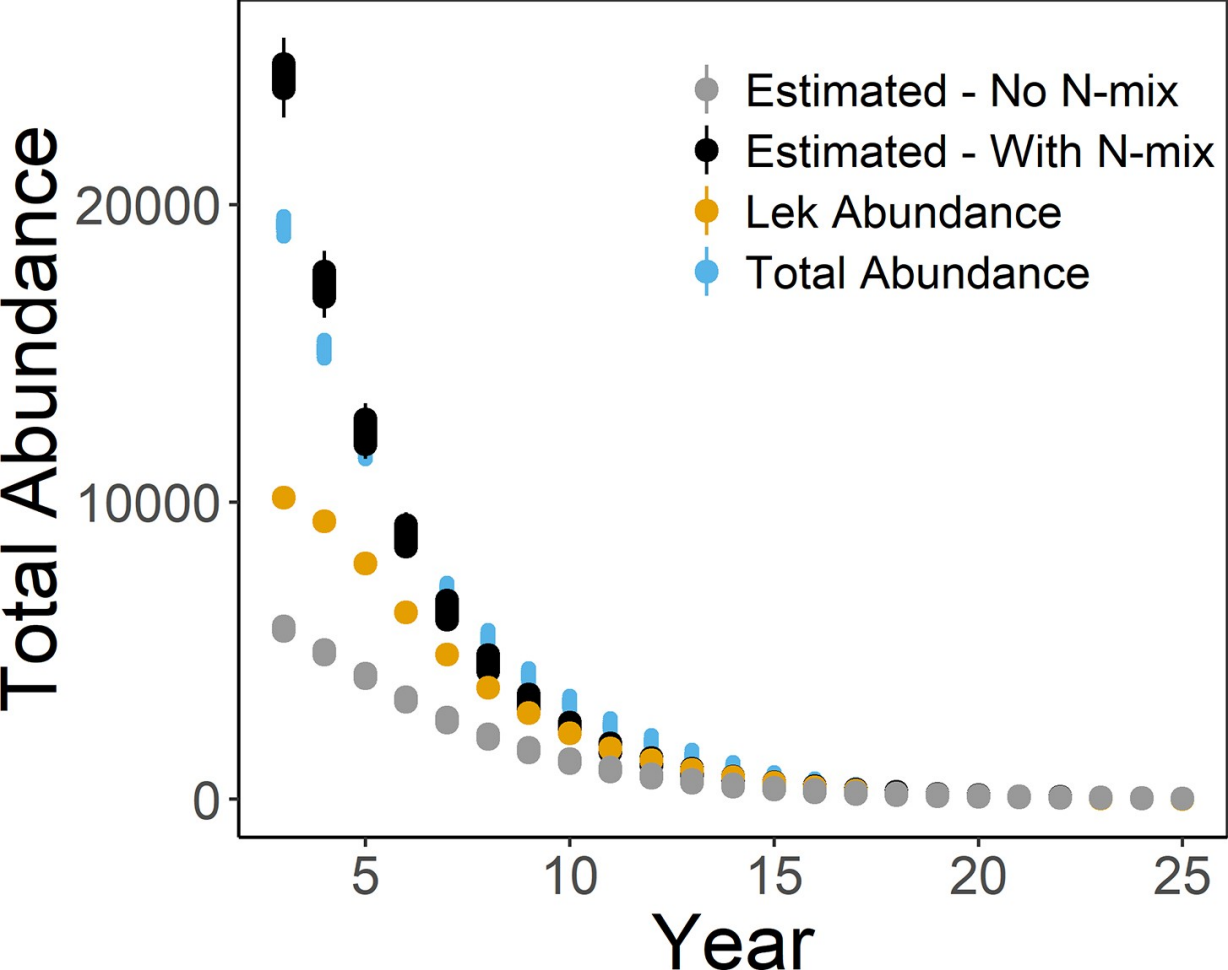
Comparison of analytical methods. Abundance of total lesser prairie-chickens (“Total Abundance”; blue) and the number of lekking lesser prairie-chickens (“Lek Abundance”; yellow) from individual-based model simulations with density-based lek attendance and detection probability analyzed with an N-mixture model (“Estimated–With N-mix”; black; Scenario 4N) and without an N-mixture model (“Estimated–No N-mix”; gray; Scenario 4). Points represent mean or true values from each simulation and whiskers represent 95% quantiles for each simulation.

Estimates of mean long-term population growth rates based on observed lek attendance indicated a decreasing population in all scenarios (95% CIs <1; Fig 2). When detection probability was 1, estimates of the mean long-term population growth rate were unbiased (Scenarios 1 & 2; Fig 3). However, when detection probability was <1, estimates of $\bar{\lambda }$ were biased high (Scenarios 3–13; Fig 3). Scenario 2 resulted in the lowest bias in $\bar{\lambda }$ while perfect lek attendance with *p* <1 had slightly higher bias (Scenario 3; Fig 3). When density affected lek attendance rates, a smaller proportion of individuals attended leks in the first years of the study (i.e., when the population was larger than the carrying capacity of the lek), resulting in larger $\bar{\lambda }$ and bias when based on lek counts (Scenario 4; Figs 2, 3 and 4). Estimated abundance did not overlap with simulated true abundance until year 14 (Scenario 4; Fig 4).

**Fig 5:**
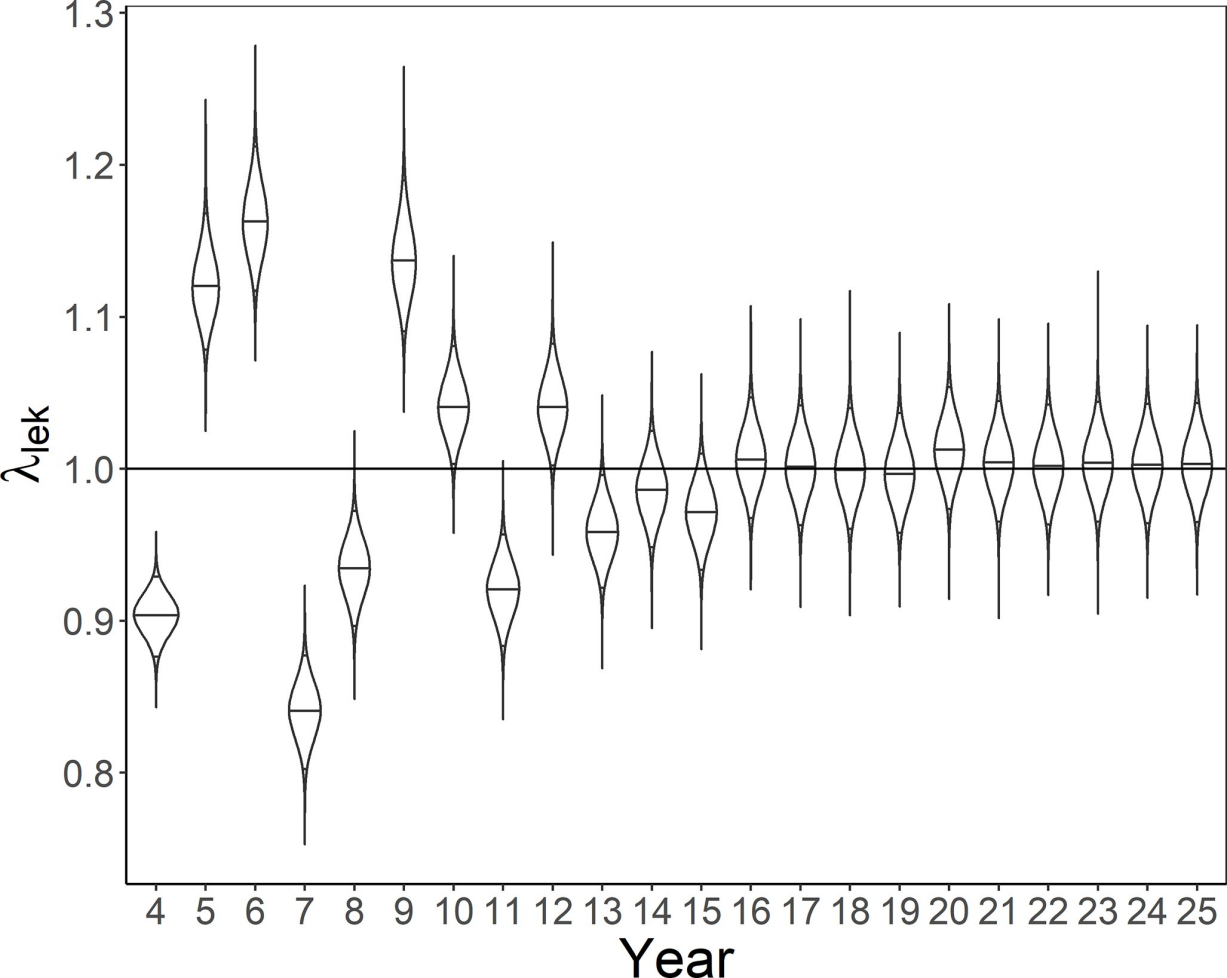
Violin plot of annual population growth rate of lesser prairie-chickens. Simulated data assuming temporally-varying detection probability (Scenario 9) from 100 simulations were estimated without an N-mixture model.

As detection probability increased from a mean of *p* = 0.25 to 0.375 and 0.5, the estimated abundance on leks increased, resulting in decreased $\bar{\lambda }$ and bias (Scenarios 5–7; Figs 2 & 3). As detection probability decreased in time, from 0.75 to 0.25 (Scenario 11), estimates of $\bar{\lambda }$ and bias decreased as well (Figs 2 & 3). While random variation in detection probability by year, site, or year and site resulted in similar bias and estimates of $\bar{\lambda }$ (Figs 2 & 3), tracking solely changes in annual population growth rate (λ_t_) resulted in apparent boom and bust dynamics (Fig 5). Overall, mean long-term population growth rate was consistently biased high for models that did not account for detection probability with the exception of a decreasing trend in detection probability (Scenario 11; Fig 3). The most variation in bias was seen when detection probability varied randomly and density-based lek attendance occurred (Scenario 13; Fig 3).

### Estimates from N-mixture model with detection probability

**Fig 6:**
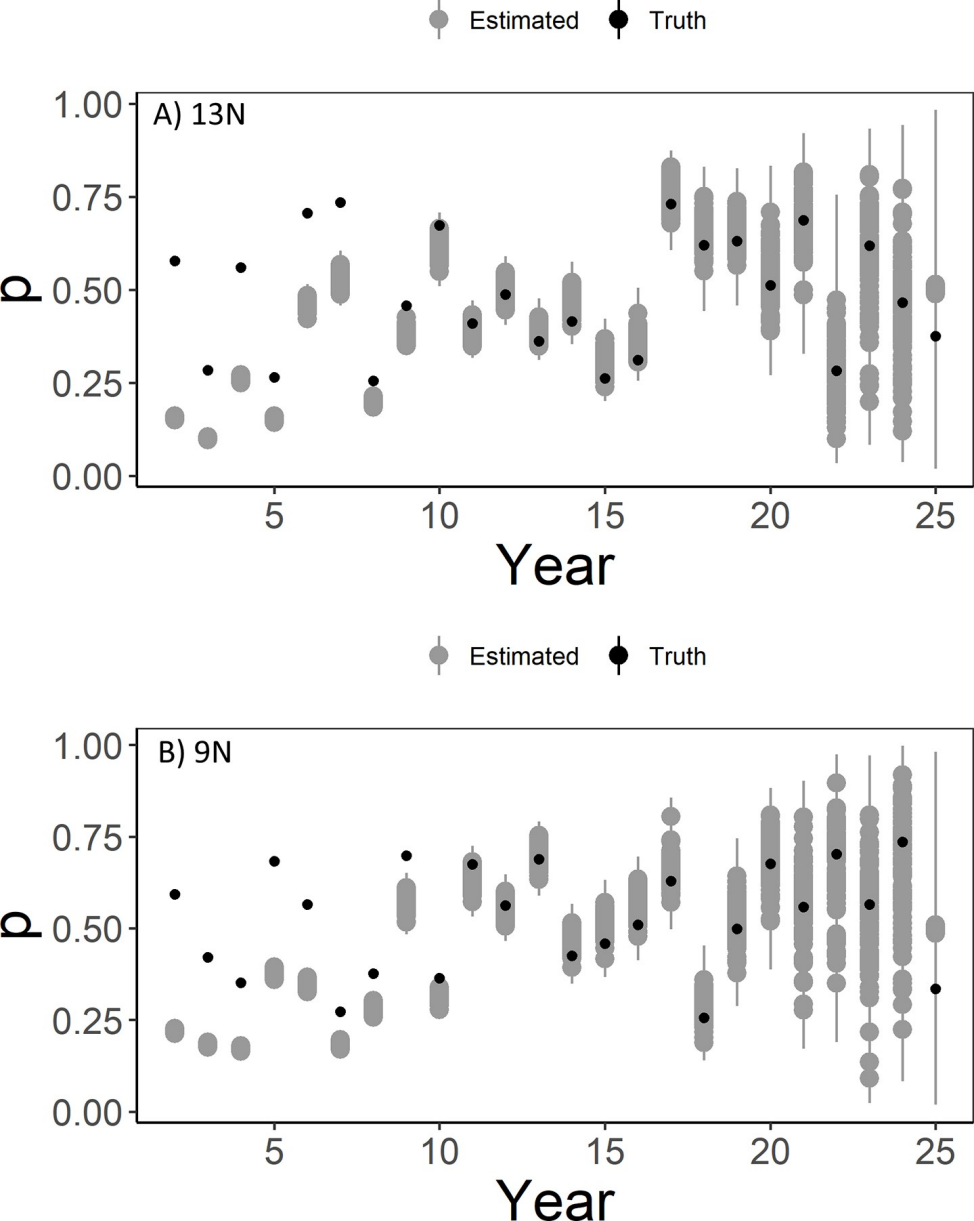
Detection probability estimates from models with density-based lek attendance. Detection probability estimates (“Estimated” in gray) of lesser prairie-chickens from an N-mixture model with density-based lek attendance (A, Scenario 13N) and without (B, Scenario 9N) and random detection probability. True values of detection probability are shown in black (“Truth”). Points represent mean or true values from each simulation and whiskers represent 95% quantiles for each simulation.

The N-mixture model resulted in negatively biased estimates of $\bar{\lambda }$ ($\bar{\lambda }$ was underestimated; Fig 3). While most of the simulations of $\bar{\lambda }$ from the N-mixture model resulted in similar posterior distributions, Scenarios 8N and 10N resulted in slightly larger posterior means of $\bar{\lambda }$ (Fig 2 & S1 Fig). Bias was slightly lower for the N-mixture model with detection randomly varied by site and year (Scenario 10; Fig 3). Variation in the bias was greatest when detection probability varied randomly and density affected lek attendance (Scenario 13; Fig 3). While estimating detection probability in the N-mixture model yielded more conservative estimates of $\bar{\lambda }$ when density affected lek attendance, total abundance of the population was overestimated in the first years of the simulation and underestimated through years 6–13 and did not overlap simulated abundance until year 14 (although bias for $\bar{\lambda }$ did not differ from other scenarios; Fig 4). The underestimation coincided with an underestimation of detection probability from years 2–9 but does not account for differences in years 10–13 (Fig 6).

**Fig 7:**
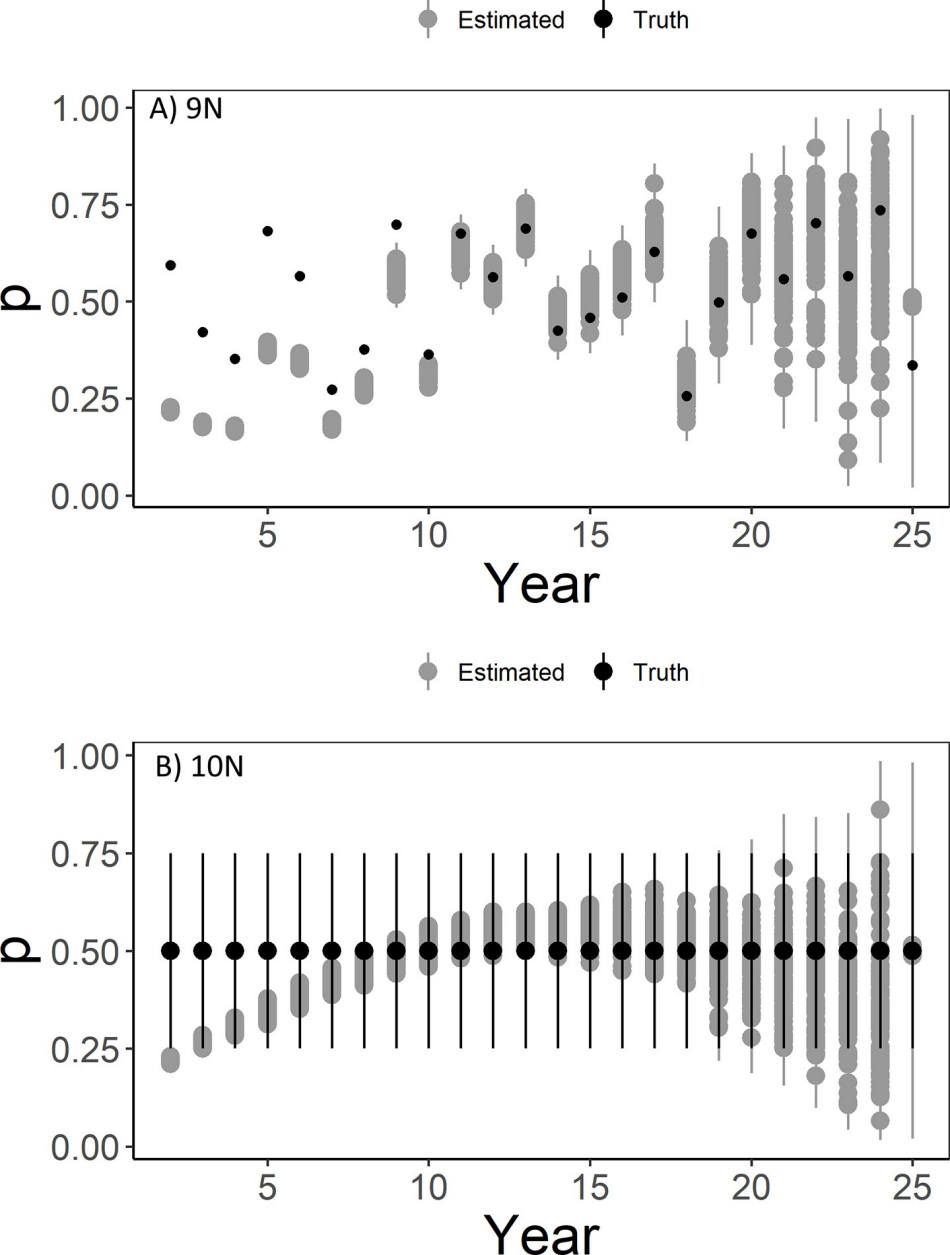
Detection probability estimates from models with temporally-varying detection probability. Detection probability estimates (“Estimated” in gray) of lesser prairie-chickens from an N-mixture model with detection probability varying randomly by time (A, Scenario 9N) and by time and space (B, Scenario 10N). True values of detection probability are shown in black (“Truth”) with true values of detection probability averaged over site for each year. Points represent mean and true values from each simulation and whiskers represent 95% quantiles for each simulation.

**Fig 8:**
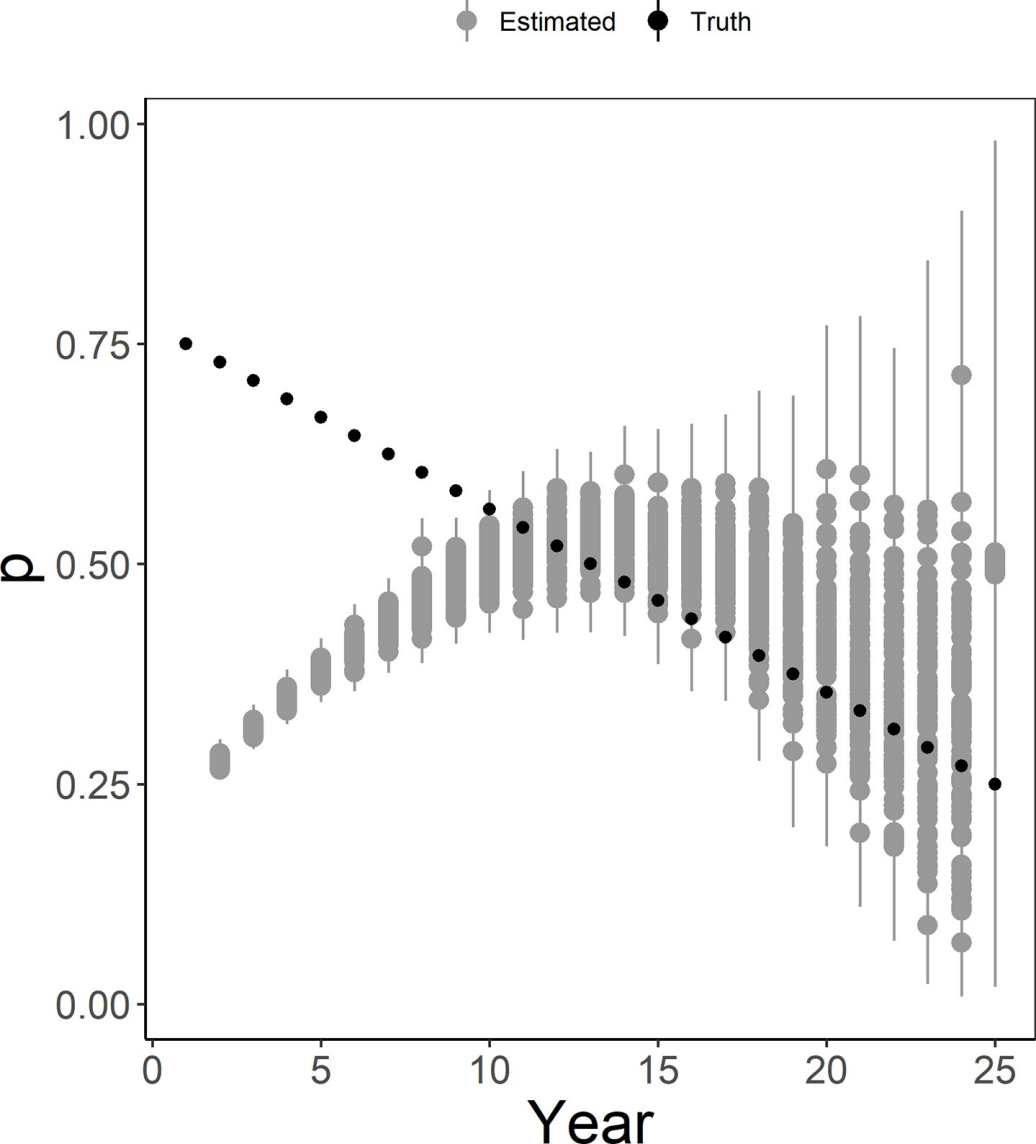
Detection probability estimates from models with decreasing detection probability. Detection probability estimates (“Estimated” in gray) of lesser prairie-chickens from an N-mixture model with detection probability decreasing by time (Scenario 11N). True values of detection probability are shown in black (“Truth”). Points represent mean or true values from each simulation and whiskers represent 95% quantiles for each simulation.

Issues related to detection probability estimation were not limited to models with density-based lek attendance, as similar issues occurred when detection probability varied by year (Scenario 9N; Fig 7), when it varied by year and site (Scenario 10N; Fig 7), or even when it was fixed over year and site at *p* = 0.5 (Scenario 7N; S2 Fig). Rather, in every scenario with the N-mixture model, *p* was underestimated during the first 5–10 years of the study with the greatest underestimation occurring when there was a decreasing trend in detection probability (Scenario 11N; Fig 8).

## Discussion

The virtual ecologist approach allows for assessment of monitoring designs based on hypothesized population and observation processes. Given the common use of lek counts for monitoring lekking species, our virtual ecologist model has wide applicability. Generally, our results indicate that monitoring using lek counts alone may not fully capture persistent long-term declines in abundance when detection probability is <1, especially when density affects lek attendance rates. When accounting for detection probability with an N-mixture model, mean long-term population growth rates were underestimated, resulting in population growth rates below true values. Neither commonly used method to estimate long-term population growth rates was able to accurately capture population abundance until the population had decreased substantially (i.e., year 14).

Estimating changes in abundance on leks based on count data led to population growth rates that were biased relative to rates estimated using female demographic rates, which has been identified as an issue in other studies using lek-based surveys to estimate annual population growth rate [8,9]. The bias of population growth rates based on lek counts is likely due to the lack of accounting for detection probability, a surveyor’s prior knowledge about the status of the lek, and temporary emigration. If detection probability is not incorporated in estimates of abundance, small changes in detection probability can lead to erroneous inference based on population growth rates [3]. In our simulations, even a relatively high detection probability of 50% resulted in biased population growth rate and a lack of concordance between true changes in population abundance and estimated abundance. Moreover, the lesser prairie-chicken has been thought to exhibit “boom and bust” population dynamics, with large fluctuations occurring from year to year based on dynamic environmental conditions [18,21]. Given results from simulations not accounting for annually-varying detection probabilities, it is unclear what proportion of the variation in population abundance is due to underlying boom and bust cycles versus observation error from lek-count data.

While accounting for detection probability can help to address bias in lek-count survey data, it does not fully account for zeros in these data due to varying lek attendance rates or individuals not attending leks at all. Our model of varying lek attendance was developed to quantify the effects of individuals that were not detected because they were not present on the survey area due to the carrying capacity of the lek being smaller than the total population available for lekking. Biologically, we would expect the carrying capacity of a given lek to remain relatively constant (e.g., 20 birds in an area with high quality lekking habitat), assuming hens are present to attend leks and lekking habitat is available annually. Given a constant carrying capacity, increases and decreases in latent abundance do not necessarily coincide with estimates of population growth rate based on leks.

The lack of correspondence between population growth rates based on leks and vital rates has important implications for monitoring lekking species, particularly lesser prairie-chickens. Our results suggest that if lek surveys based on males are used for population inference, monitoring annual changes in population growth rates may not fully capture mean long-term population growth rates ($\bar{\lambda }$). If the goal of management actions for lekking species are directed at improving female survival or reproductive output, then using lek-based surveys for monitoring the results of management actions may not be the most effective way to detect changes in population growth rates. Rather, lek-based surveys are more appropriate for long-term inference related to density of leks specifically [10] when detection probability is incorporated, and may not always correlate with changes in the total population, although leks are still important centers for female survival and reproduction [29].

While inference based on our individual-based model to all lekking avian species may be limited, if other lekking species have lek attendance rates <1 or temporally-varying detection probability, estimating true abundance of males on a lek may be difficult. These two limitations present challenges for monitoring lesser prairie-chickens, and more generally lekking species where lek attendance rates <1, given that lesser prairie-chickens and other lekking grouse species typically exhibit lek attendance rates <1 [30–33]. Current methods to estimate range-wide abundance of lesser prairie-chickens do not include lek attendance rates in the calculations and assume constant detection probability among years [20,34]. Given lek attendance rates <1, our results suggest that true abundance of lesser prairie-chickens could be larger than current lek population estimates. While the true population may be larger than current estimates, high variance associated with uncertainty arising from imperfect detection and lek attendance rates <1 makes identifying changes in population growth rates based solely on counts of males on leks difficult.

Using simulation studies can reveal important assumptions about monitoring programs [5]. Our individual-based model allowed us to better incorporate stochasticity into the underlying process governing population change based on individual survival and reproduction, especially when paired with an observation process [35]. However, our model was not spatially explicit, and not a true representation of the complexity of the system. Movement among leks may further confound abundance estimates for lekking species [11]. Our estimates of female survival or nesting rates could potentially be biased low due to transmitter or nest monitoring effects, but research thus far has not found differences in female survival due to transmitters [36], and nests were only flushed once or when monitored females left the nest. Flushing greater prairie-chickens (*Tympanuchus cupido*) results in the majority (95%) of females returning to nests and nest success is not negatively affected [37]. Our results indicate that lek counts can result in biased population growth rate estimates and monitoring lesser prairie-chicken leks in conjunction with vital rates would likely provide better inference about changes in population growth rate. In the best-case scenario for managers and biologists, lek-based surveys are conducted with repeated counts to account for detection probability, and lek attendance is nearly 1. Our results indicate that it may take surveys years to accurately estimate abundance despite consistent monitoring even if detection probability is included in the survey analysis. Additionally, if survey programs want to obtain unbiased estimates of abundance of male lesser prairie-chickens, then additional methods for estimating lek attendance rates (e.g., radio transmitters) could be incorporated into the monitoring protocol to improve estimates. Alternatives to using lek counts for abundance estimates could be presence of leks in response to management actions, broad-scale monitoring for range shifts of lek-based species, or presence/absence of leks across a landscape. The objectives of the monitoring program could be assessed in an adaptive management framework to balance tradeoffs between costs and information gained.

## Supporting information

S1 Fig Mean and 95% CIs for output of simulated lesser prairie-chickens from Scenario 7N & 8N (left) and 7N & 10N (right).
(TIF)

S2 Fig Estimated detection probabilities of simulated lesser prairie-chicken populations over a 25-year period. Black points represent true values of detection probability (p = 0.5; Scenario 7) while gray represents estimates from N-mixture model results with mean (points) and 95% quantiles (whiskers) from each simulation.
(TIF)

S1 File R code for individual-based model. (TXT)
