# Supplementary figures and images for: Using an individual-based model to assess common biases in lek-based count data to estimate population trajectories of lesser prairie-chickens
Source: PLoS One. 2019 May 17;14(5):e0217172. doi: 10.1371/journal.pone.0217172 (PMC6524812; doi:10.1371/journal.pone.0217172)

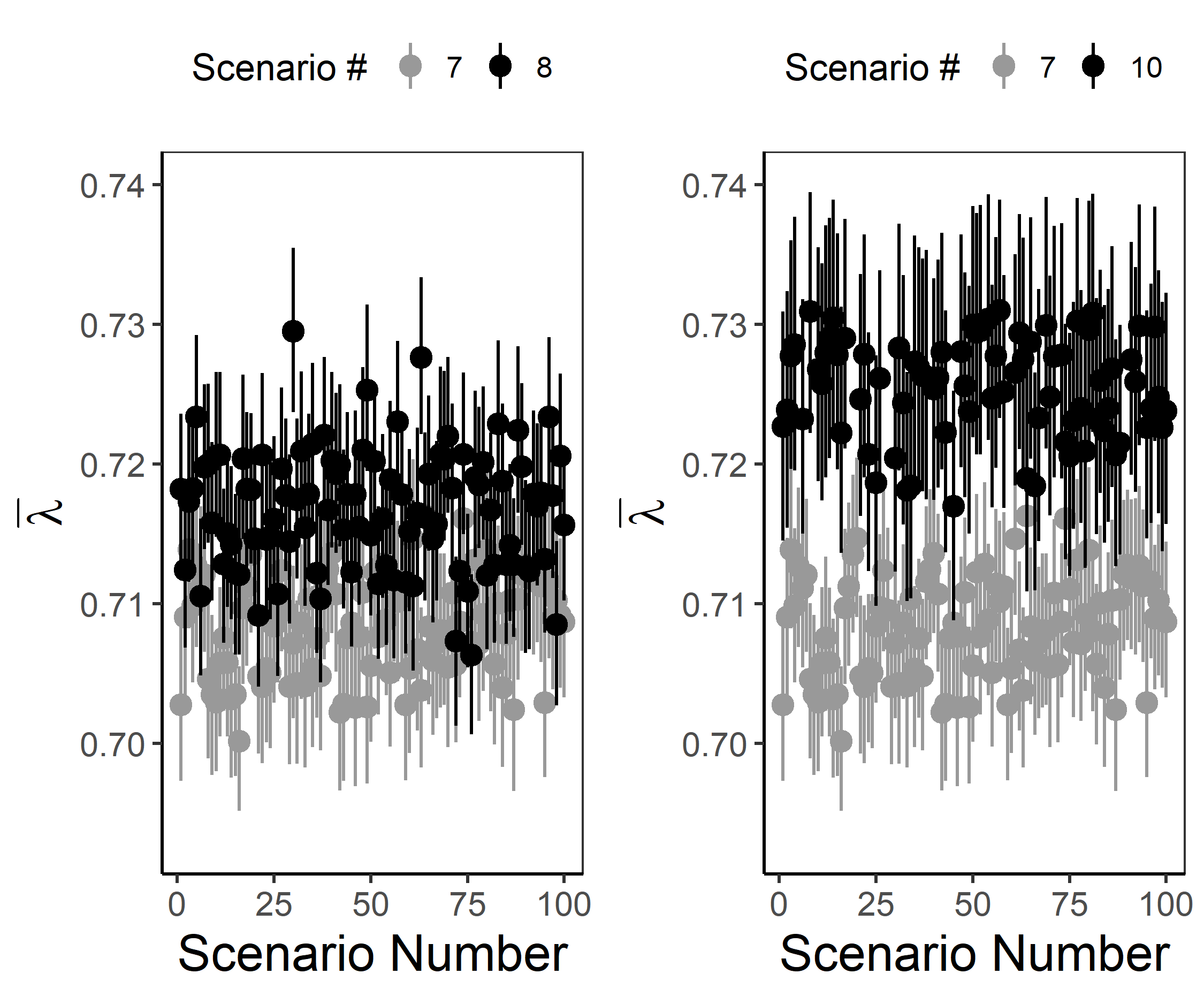

Supplement: S1 Fig — Mean and 95% CIs for output of simulated lesser prairie-chickens from Scenario 7N & 8N (left) and 7N & 10N (right). (TIF) [file pone.0217172.s001.tif]

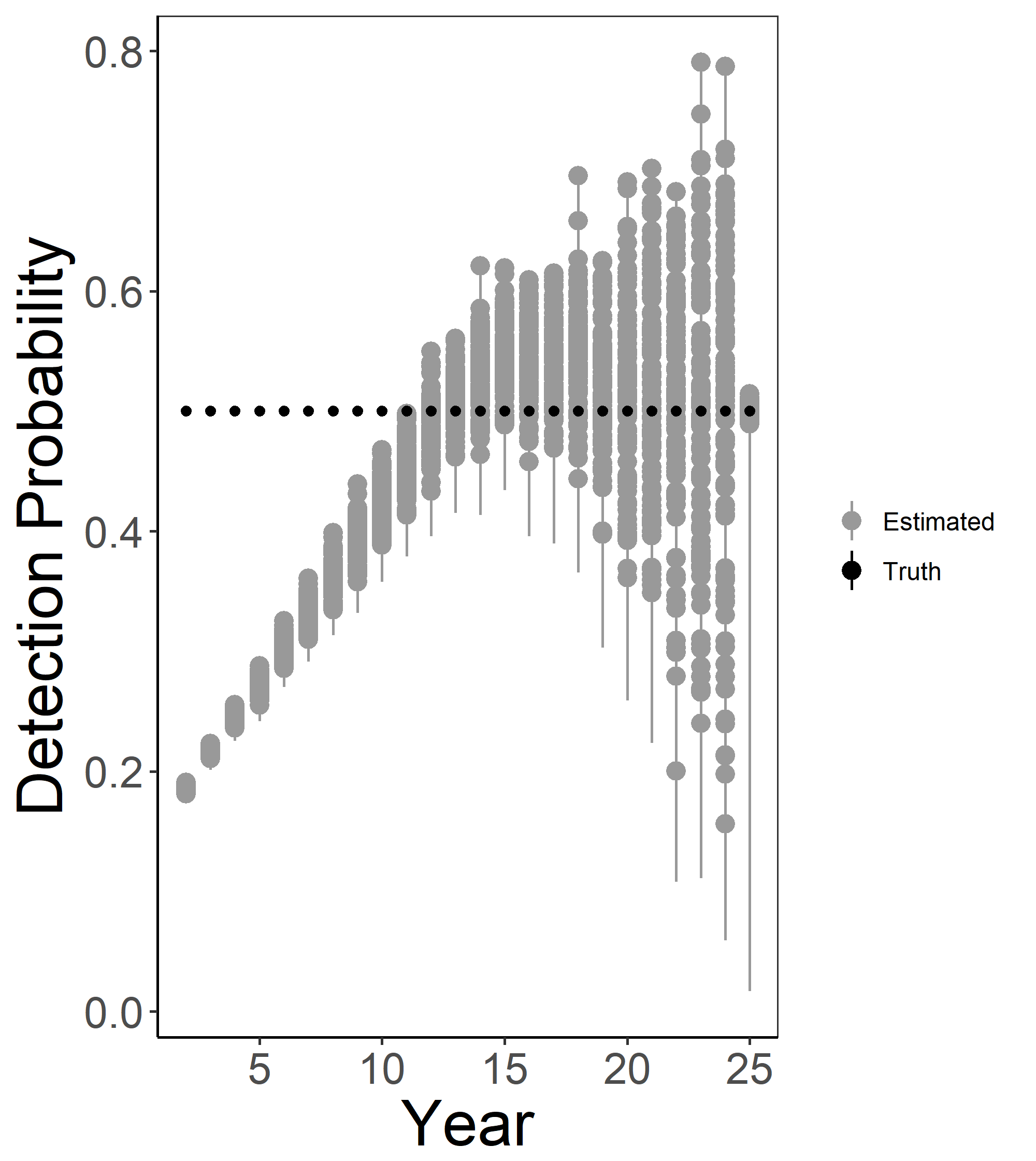

Supplement: S2 Fig — Black points represent true values of detection probability (p = 0.5; Scenario 7) while gray represents estimates from N-mixture model results with mean (points) and 95% quantiles (whiskers) from each simulation. (TIF) [file pone.0217172.s002.tif]
